# Actions of Agonists, Fipronil and Ivermectin on the Predominant *In Vivo* Splice and Edit Variant (RDL_bd_, I/V) of the *Drosophila* GABA Receptor Expressed in *Xenopus laevis* Oocytes

**DOI:** 10.1371/journal.pone.0097468

**Published:** 2014-05-13

**Authors:** Kristin Lees, Maria Musgaard, Siros Suwanmanee, Steven David Buckingham, Philip Biggin, David Sattelle

**Affiliations:** 1 Faculty of Life Sciences, University of Manchester, Manchester, United Kingdom; 2 Department of Biochemistry, University of Oxford, Oxford, United Kingdom; 3 Wolfson Institute for Biomedical Research, Department of Medicine, University College London, London, United Kingdom; Biogen Idec, United States of America

## Abstract

Ionotropic GABA receptors are the targets for several classes of insecticides. One of the most widely-studied insect GABA receptors is RDL (resistance to dieldrin), originally isolated from *Drosophila melanogaster*. RDL undergoes alternative splicing and RNA editing, which influence the potency of GABA. Most work has focussed on minority isoforms. Here, we report the first characterisation of the predominant native splice variant and RNA edit, combining functional characterisation with molecular modelling of the agonist-binding region. The relative order of agonist potency is GABA> muscimol> TACA> β-alanine. The I/V edit does not alter the potency of GABA compared to RDL_bd_. Docking calculations suggest that these agonists bind and activate RDL_bd_I/V through a similar binding mode. TACA and β-alanine are predicted to bind with lower affinity than GABA, potentially explaining their lower potency, whereas the lower potency of muscimol and isoguvacine cannot be explained structurally from the docking calculations. The A^301^S (resistance to dieldrin) mutation reduced the potency of antagonists picrotoxin, fipronil and pyrafluprole but the I/V edit had no measurable effect. Ivermectin suppressed responses to GABA of RDL_bd_I/V, RDL_bd_ and RDLbdI/VA301S. The dieldrin resistant variant also showed reduced sensitivity to Ivermectin. This study of a highly abundant insect GABA receptor isoform will help the design of new insecticides.

## Introduction

GABA-gated chloride channels (GABA_A_Rs) are of considerable interest in neuroscience, medicine and drug discovery. This is due to several factors. First, they are widely distributed throughout the nervous systems of vertebrates [1], [2] and invertebrates [3]–[5]. Secondly, they are the targets of a large number of diverse drugs, including anxiolytic and anticonvulsant benzodiazepines [6] as well as anaesthetics such as propofol [7] and steroids [8]. Thirdly, they are targets of several classes of commercially-important insecticides, such as cyclodienes (including dieldrin) and fipronil [4], while in nematodes they are targeted by piperazine [9]. A better understanding of the molecular basis of GABA_A_R function will therefore impact strongly on several areas of neuroscience.

The major GABA_A_R of the fruit-fly, *Drosophila melanogaster,* is the RDL (resistance to dieldrin) subunit [10], [11], which has played an important strategic role in our understanding of how ligands regulate GABA_A_Rs. Not only is it from a model organism with the most extensive genetic toolkit, but the receptor (unlike nearly all vertebrate GABA_A_Rs) readily expresses robust, functional homo-oligomers, assisting the interpretation of structure-function studies and molecular simulation studies aimed at understanding how ligands interact with the receptor. RDL was the first invertebrate GABA receptor to be cloned and functionally expressed, initially from the fruit fly [10], [11], and subsequently from many other insects [12]–[14]. In most insects, cyclodiene resistance is conferred by an alanine-to-serine substitution, but an alanine-to-glycine replacement has also been observed [15]. Identical resistance alleles have been found in geographically distant regions pointing towards multiple origins of resistance [16]. This RDL A^301^S mutation results in high levels (>4000-fold) of resistance to the cyclodiene dieldrin [17] and the structurally-related plant compound picrotoxin, a mixture of the two compounds picrotoxinin and picrotin.

More importantly, *Drosophila* RDL has also been intensively studied as a model of the effects of mRNA editing and splicing – major sources of phenotypic diversity outside the genome. However, studies to date suffer from a major weakness, in that they are performed only on rare edit/splice isoforms [18]. This paper aims to address this problem.

Alternative splicing in two locations of RDL yields two variants each for exons 3 (variants a and b) and 6 (variants c and d). All four possible splice isoforms are transcribed [10]. This alternative splicing affects agonist potency [19]. In addition, *Rdl* undergoes A-to-I RNA editing [20] which leads to the substitution of four amino acid residues in functionally significant regions: R122G (extracellular N-terminal), I283V (TM1), N294D (intracellular TM1–TM2 loop) and M360V (intracellular TM3–TM4 loop). The combination of alternative splicing and RNA editing generates considerable variation in the physiology and pharmacology of RDL. For example, Es-Salah et al. [21] showed that the edit site R122G, located between ligand binding loops D and A, affects agonist potency and reduces sensitivity to fipronil. Furthermore, it is interesting to note that Fisher [22] found that the large intracellular loop between TM3 and TM4, which contains the site of the M360V edit, influences GABAR desensitisation kinetics and GABA sensitivity.

In a comprehensive analysis of the impact of RNA A-to-I editing of *Drosophila* RDL in different splice backgrounds, Jones et al. [18] showed that alternative splicing and RNA editing have a combined effect on GABA potency and the extent of RNA editing varies between splice variants and also depends on the developmental stage. The bd isoform was the predominant splice variant with transcript levels 26 times greater than the ad form (the second most abundant variant) and it was the only splice variant to be edited at all four sites. Of the four edits, I283V was the most abundant across all life stages. However, despite being the commonest variant and therefore the most abundant form of RDL exposed to insecticides, its physiology and pharmacology are not well studied. Prior to appreciation of the potential impact of editing and splicing, most studies used the RDL_ac_ variant which has been shown to be the least abundantly expressed of the four splice variants. Here we provide the first detailed characterisation of heterologously expressed RDL_bd_I/V, the most abundant native form of the *Drosophila* RDL GABA receptor.

## Materials and Methods

The RDL_bd_ splice variant with and without the I283V edit [18] was the starting point for site-directed mutagenesis. The Quik Change Site-Directed Mutagenesis Kit (Stratagene) was used to introduce an alanine to serine mutation (*gcgctc* to *tcgctc*) into the Rdl_bd_I283V (Rdl_bd_I/V) variant to produce the dieldrin-resistant mutations Rdl_bd_I283VA^301^S (Rdl_bd_I/VA^301^S). To help the reader differentiate between a natural editing site and a mutation we have put the mutation in superscript. The sequences of the forward and reverse primers were:

5′ CAACGCCGGCGCGTGTG**T**CGCTCGGTGTGACAACC 3′ (sense)

5′ GGTTGTCACACCGAGCG**A**CACACGCGCCGGCGTTG 3′ (antisense)

(altered nucleotide in bold). The presence of the mutation was confirmed by sequencing. Templates were linearised with *Xba*I, and capped RNA was synthesised using the T7 mMessage Machine Kit (Ambion).

### Two-electrode Voltage-clamp Electrophysiology

Stage V-VI oocytes from *Xenopus laevis* were purchased from the European Xenopus Resource Centre at the University of Portsmouth and defolliculated manually after a 1 h incubation with collagenase type 1A (2 mg/ml) (Sigma) and injected with 50 ng (at 1 ng/nl) cRNA encoding RDL_bd_, RDL_bd_I/V or RDL_bd_I/VA^301^S. Oocytes were incubated in filter-sterilized, standard oocyte saline (SOS) consisting of: 100 mM NaCl, 2 mM KCl, 1.8 mM CaCl_2_, 1 mM MgCl_2,_ 5 mM HEPES (pH 7.6) and supplemented with 100 U/ml penicillin, 100 µg/ml streptomycin, 50 µg/ml gentamicin and 2.5 mM sodium pyruvate at 18°C. Currents were recorded 24–48 h post-injection. Oocytes from at least 3 animals were used for each experiment.

Xenopus oocytes were voltage clamped at −100 mV under 2-electrode voltage clamp. Only oocytes which yielded stable responses to at least three control concentrations of GABA applied at 2 min intervals were used. Agonists were obtained from Sigma (UK) and dissolved in SOS. Concentration-response curves were constructed by challenging the oocyte to increasing concentrations of agonist for 5 s at a flow-rate of 4 ml/min with at least 2 min between challenges to reduce the effects of desensitisation. Responses were normalised to a maximal GABA-evoked response.

Picrotoxin (PTX) (Sigma, UK), ivermectin (IVM) (Sigma, UK), fipronil and pyrafluprole (gifts from Dr Lance Hammerland, Merial Animal Health) were first dissolved in dimethylsulphoxide (DMSO) and then diluted in SOS to the required concentrations. Responses to GABA were not affected by DMSO at its final concentration (1% v/v). To determine inhibitory activity, the antagonists were added after three successful control applications of 1 mM GABA. Oocytes were pre-treated with the antagonists for 3 min followed by co-application of antagonist and GABA for 5 s.

### Data Analysis

Data are presented as mean ± SEM of three to five independent experiments. Agonist oocyte data were normalised to the maximal current obtained from that oocyte and non-linear regression was used to fit the concentration-response data for each oocyte obtain the pEC_50_ values, fitting the normalised data to a 3-parameter sigmoidal function:
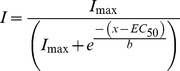
where *I* is the normalized current, *I_max_* is the maximum current evoked by that compound, *x* is the concentration, EC_50_ is the concentration that evokes half the maximum current, and *b* is the slope. To obtain the concentration-inhibition relationship, the response after co-application of agonist and antagonist was normalised to the control response against GABA and analysed to obtain the IC_50_ value. Statistical tests were performed using a 1-way ANOVA on the EC_50_s with post-hoc *t*-tests with a significance level of P<0.05.

### Homology Modelling

Models of RDL_bd_ with I283V and additionally R122G and N294D were constructed using MODELLER 9.9 [23] based on the structure of the *Caenorhabditis elegans* GluClα glutamate-gated chloride channel [24]. The sequence alignment was generated using ClustalW [25], [26]. The best models were selected based on the MODELLER objective function, the DOPE score and PROCHECK Ramachandran plot statistics. R122G and N294D are both located more than 20 Å from the agonist binding site and will thus presumably have no influence on the binding models.

### Agonist Docking

The five agonists, GABA, TACA, β-alanine, isoguvacine and muscimol, were constructed in Maestro version 9.3 (Schrödinger, LLC, New York, NY, 2012 academic version). All agonists were docked in their zwitterionic form in which they are expected to be found at physiological pH [27]–[30]. Since Autodock Vina does not account for potential flexibility in ring structures, five different ring conformations for isoguvacine were generated in Maestro.

Protein and ligands were prepared for docking using AutoDock Tools [31] and docking calculations were performed with AutoDock Vina [32]. The centre of the search box was defined from the centre of mass of E204 (chain E) and R111 (chain A), and the size of the box was 17×17×20 Å^3^. A maximum of 20 generated binding models for each input structure was requested with a maximum energy difference between the best and worst binding models of 5 kcal/mol. The sidechains of E204 (chain E) and R111 (chain A), in each end of the binding pocket, were treated as flexible in the ligand docking.

### Statistical Analyses

All numerical data are presented as mean ± standard error of the mean. Statistical tests of significance are given in the text.

### Ethics

Since no experiments were conducted on living animals or on humans, ethics approval for this study is not required.

## Results

### Agonist Profile – functional and Modelling Studies

**Figure 1:**
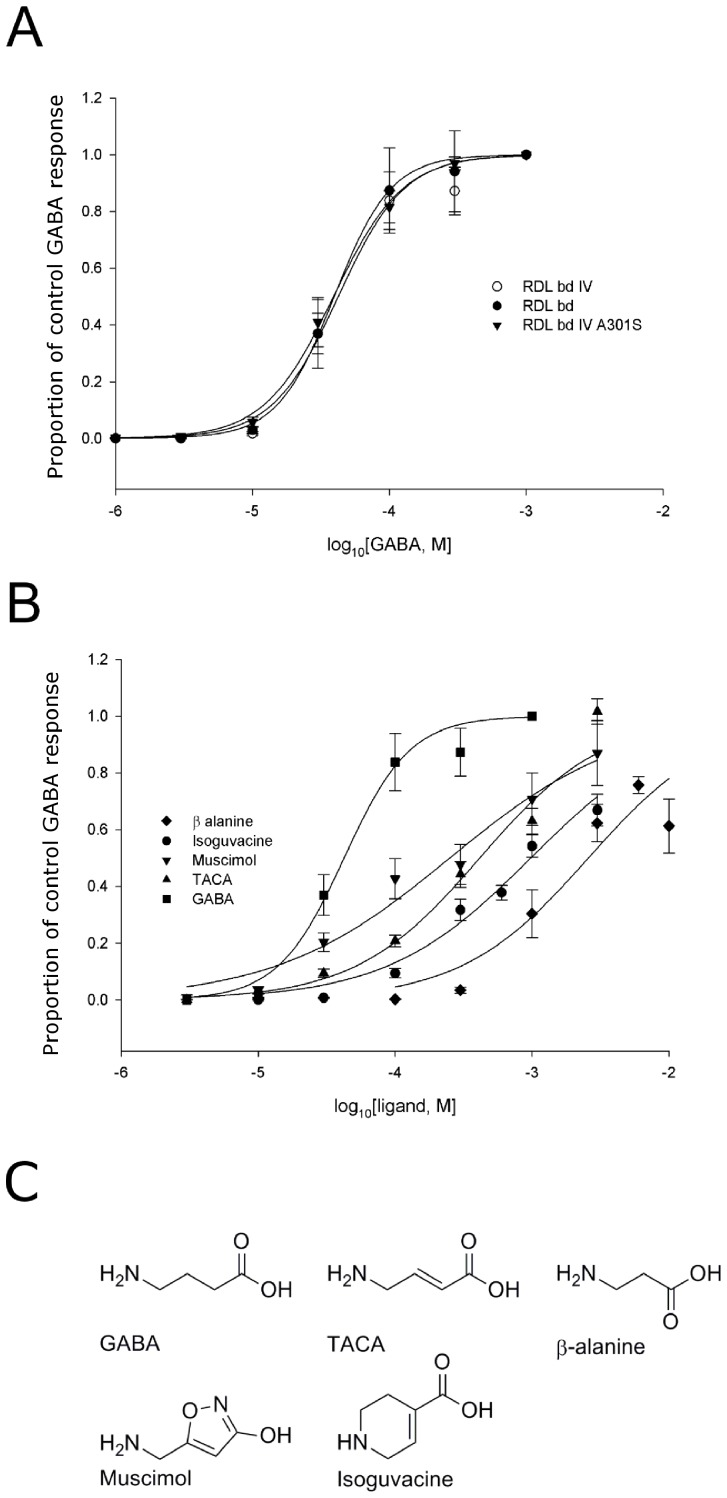
Concentration response curves for GABA and analogues mediated by variants of the RDL_bd_ splice variant. (A) Concentration response curves for the three RDL subunits: RDL_bd_, RDL_bd_IV and RDL_bd_IVA^301^S to GABA (structure inserted). Data were normalised to 1 mM GABA. Each data point is the mean ± S.E.M from four to six oocytes from at least three separate frogs. Representative responses to 100 µM GABA are shown for each variant. Scale bars indicate 0.5 µA, 1 s. (B) Concentration response curves showing responses elicited by GABA and analogues at RDL_bd_IV receptors in Xenopus oocytes. GABA is the most potent agonist, and the rank order is GABA> muscimol> TACA>>β-alanine. Isoguvacine did not reach saturation. Data were normalised to the response to 1 mM GABA. Each data point represents mean±S.E.M from five to eight oocytes. (C) The chemical structures of the agonists tested in the present study: GABA (γ-amino butyric acid), TACA (trans-4-aminocrotonic acid), β-alanine, muscimol and isoguvacine.

Oocytes expressing RDL_bd_, RDL_bd_I/V or RDL_bd_I/VA^301^S produced concentration-dependent inward currents in response to bath-applied GABA over the range 10^−6^ to 10^−3^ M (Fig. 1A). We could not obtain saturation of responses to muscimol, isoguvacine or TACA within the limits of solubility (see Fig. 1B). Nonetheless, fits to the curves allowed the EC_50_s to be estimated and there is no statistically significant difference (F(2,95) = 1.3, P = 0.27) for GABA pEC_50_s between the three RDL_bd_ subtypes (RDL_bd_: 4.24±0.09, RDL_bd_I/V: 4.34±0.07, and RDL_bd_I/VA^301^S:4.34±0.06). Maximum current amplitudes were recorded above ∼1 mM GABA.

Five GABA analogues (GABA, TACA, β-alanine, muscimol and isoguvacine) were tested for agonist activity on RDL_bd_I/V (Fig. 1). Muscimol, TACA and β-alanine induced concentration-dependent inward currents. Responses to isoguvacine did not saturate at 3 mM and produced non-specific responses at 10 mM in uninjected oocytes. Responses to TACA, muscimol and β-alanine also failed to saturate at 6 mM, 3 mM and 10 mM respectively, and were insoluble at higher concentrations. It is therefore not clear where the true saturation concentration is, so estimates of pEC_50_ are therefore less trustworthy. With the above caveat regarding the failure to reach saturation, fits to the concentration/response curves suggest that the relative order of agonist potency of the RDL_bd_I/V isoforms is as follows (pEC_50_s in parentheses): GABA (4.42±0.05)>muscimol (4.02±0.04) >TACA (3.58±0.04)>β-alanine (2.51±0.03). A 1-way ANOVA revealed that these differences are statistically significant (F(4,255) = 274, P<0.0001) and post-hoc unpaired *t*-tests indicated that pEC_50_s of muscimol (P<0.001), TACA (P = 0.001), and β-alanine (P = 0.0012) were all significantly different to that of GABA.

We next set out to establish whether molecular docking simulations might explain the agonist profile of this receptor. TACA and isoguvacine share the GABA skeleton with an amine group and a carboxylic acid group connected by three carbon atoms (excluding the carbon atom in the carboxylic acid group). Muscimol contains a similar skeleton with a hydroxyl group instead of the carboxylic acid group. β-alanine has both the amine group and the carboxylic acid group but only two connecting carbon atoms. These structural similarities (See Fig. 1C) suggest that the five agonists might share a similar binding mode with overlapping positions of the amine and the acid except for β-alanine, which is possibly too short to span the distance required between the two groups.

Earlier modelling studies for the GABA_C_ receptor predict that GABA in this receptor forms salt bridges to R104 (R111 in RDL_bd_) and to E194 and E196 (E202 and E204 in RDL_bd_) [33], [34]. For RDL, F206 and Y254 are believed to form cation-π interactions with GABA whereas F146 does not seem to contribute to cation-π interactions [35].

**Figure 2:**
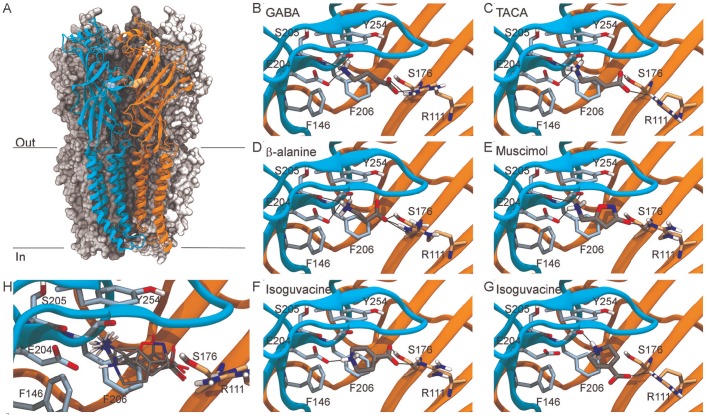
Binding models for the different agonists. (A) Overall structure of the homology model of RDL_bd_. Chains A and E are shown in cartoon representation, A in orange and E in cyan. Chains B, C and D are shown in spacefill representation in three different shades of grey. The agonist-binding site is indicated by the sidechains of R111 in chain A (light orange spacefill) and D204 in chain E (light cyan spacefill). (B–G): binding models for the docked agonists. The agonists are shown with grey carbon atoms, and relevant protein sidechains from chain A and chain E are shown with light orange and light blue carbon atoms, respectively. Only polar hydrogen atoms on agonists and sidechains are included. Potential hydrogen bonds (<3 Å) between ligand and protein are marked with black lines. (H) Superposition of the binding models in (B–F). The conformations of R111 and E204 shown are from the GABA binding model.

To explore whether a similar binding mode for the different ligands could be identified, the five agonists were docked into the agonist binding site in the extracellular domain of a homology model of the RDL_bd_ receptor. The residues R111 and E204 were used to define the binding pocket (Fig. 2). For each ligand, a variety of binding modes was generated, both inside and outside the binding site. The analysis was focused on binding modes inside the binding site, where the agonists would possibly make interactions with R111, E202 or E204, F206 and/or Y254 as suggested by previous studies for GABA.

The two best-scoring binding models of GABA (both with a predicted affinity of −5.0 kcal/mol; third-best affinity is −4.7 kcal/mol) are oriented in the same way. They illustrate a binding mode in which the carboxylate of GABA interacts with R111 through hydrogen bonds and ionic interactions, and where the amine interacts through hydrogen bonds and ionic interactions with E204 and forms hydrogen bonds to backbone oxygen atoms on S205 and F206 (Fig. 2B). In the best-scoring model, Y254 is best positioned for cation-π interactions, but also the side chains of F206 and F146 are within 4.5 Å of the positively charged amine group. This is the best scoring binding model, and additionally it fits well with earlier discussed data regarding interactions with R111, E204, Y254 and partly F206. Hence, this model is chosen as a model for GABA binding in RDL_bd_.

For TACA, which is very similar to GABA except for the presence of a double bond in the carbon chain connecting the carboxylate and the amine, the three generated binding modes with the highest affinity are positioned very similar to the chosen binding model for GABA (Fig. 2C). These three binding models with the carboxylate pointing towards R111 and the amine towards E204, and thus partly sandwiched between F206 and Y254, all have predicted affinities of −4.7 kcal/mol. The fourth-best model has an affinity of −4.0 kcal/mol. The range of interactions possible for this binding mode is more or less the same as for GABA itself, except that the TACA carboxylate group additionally forms a hydrogen bond to the S176 hydroxyl group.

**Figure 3:**
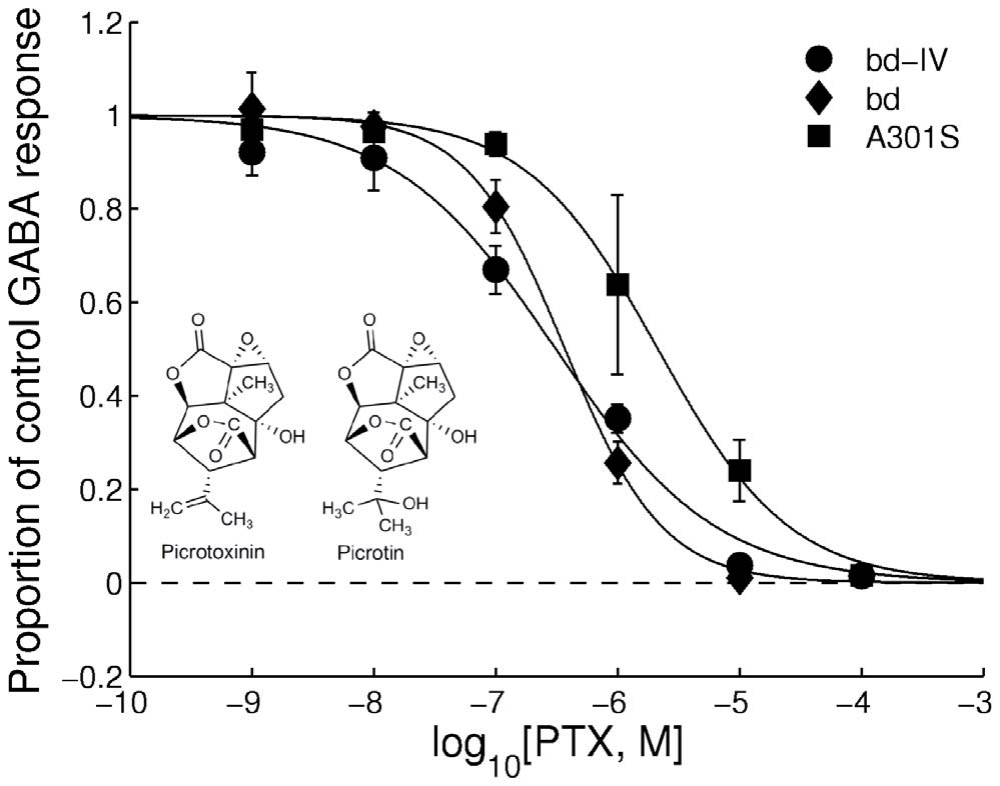
Concentration-inhibition curves showing responses elicited by PTX to 1 mM GABA in RDL, RDL_bd_IV and RDL_bd_IV/A^301^S. Each data point was normalised to the amplitude of the response to 1 mM GABA and represents the mean amplitude of the responses recorded in 7–13 oocytes from at least 3 separate batches. Structures of the two compounds found in picrotoxin (PTX), picrotoxinin and picrotin, are shown.

Despite the lack of a carbon atom in the skeleton of β-alanine relative to GABA and TACA, the three binding models for β-alanine with the highest affinities are positioned similar to the chosen binding model for GABA (Fig. 3D). The amine group superimposes with the one in GABA, and the R111 sidechain is rearranged to allow interaction with the carboxylate of the smaller β-alanine molecule. A hydrogen bond to S176 also seems likely. The predicted affinities for these three binding modes are −4.3 kcal/mol, −4.2 kcal/mol and −3.9 kcal/mol. The affinity for the fourth best model is −3.6 kcal/mol.

The binding model for muscimol with the largest predicted affinity is overall oriented in the same way as the chosen GABA binding model with the amine pointing towards E204 and the negatively charged oxygen atom pointing towards R111 (Fig. 2E). The amine forms the same interactions as the amine in GABA, while the negatively charged oxygen atom interacts with R111. The predicted affinity for this binding mode is −5.2 kcal/mol while the next best one is −5.1 kcal/mol.

Different starting conformations were used for isoguvacine as AutoDock Vina does not automatically vary the conformation of flexible rings during the docking. Thus, more poses were generated than for the other docked ligands. The best ranked binding models for each conformation can be clustered into two different binding modes, one in which the overall orientation is similar to the GABA binding model (Fig. 2F) and another, more or less rotated 90° around an axis perpendicular to the 6-membered ring, where the carboxylate group is still pointing towards R111, but the amine points directly towards backbone oxygen of F206 (Fig. 2F). Such an overall orientation is also seen for some binding modes for the other ligands, although binding modes with this orientation have a lower predicted affinity compared to modes with the amine pointing towards E204. For isoguvacine, the predicted affinity for the best model from each of the two clusters is −5.5 kcal/mol for the less GABA-like mode and −5.2 kcal/mol for the GABA-like mode. For the GABA-like binding mode, the amine interacts with E204. No other hydrogen bonds from the amine seem possible, and it is not very well positioned for forming cation-π interactions though the sidechains of Y254 and F146 are within 4.5 Å of the amine. The carboxylate interacts with R111 and S176. For the other binding mode (Fig. 2G) which is predicted to have a higher affinity, the carboxylate again interacts with R111, whereas the amine is positioned quite well for cation-π interactions with both Y254 and F206 and also forms a hydrogen bond to the backbone oxygen of F206. Judging from the scores alone, the less GABA-like binding mode (Fig. 2G) appears most physiologically relevant, whereas the GABA-like mode (Fig. 2F) seems attractive as it fits better with the proposed binding modes for the other agonists (as the comparison in Fig. 2H illustrates).

### Actions of Picrotoxin, Fipronil, Pyrafluprole and Invermectin

Given the importance of insect GABA_A_Rs in pest control, and the paucity of studies on the overwhelmingly predominant isoform of RDL, we next considered the antagonist profile of RDL_bd_I/V. The amplitude of GABA-evoked currents mediated by RDL_bd,_ the edited RDL_bd_I/V and the A^301^S point mutated RDL_bd_I/VA^301^S were reduced by bath-applied PTX in a concentration-dependent manner (Fig. 3), with pIC_50_ values estimated from fitting the dependency of inhibition on PTX concentration to be 6.46±0.18, 6.44±0.13 and 5.67±0.19, respectively. These values were statistically significantly different (F(2,60) = 5.49, P<0.001). While 1 µM PTX reduced GABA responses mediated by wild-type and RDL_bd_I/V receptors by more than 50%, the same concentration of PTX reduced responses of the A^301^S only by about 30% (Fig. 3).

**Figure 4:**
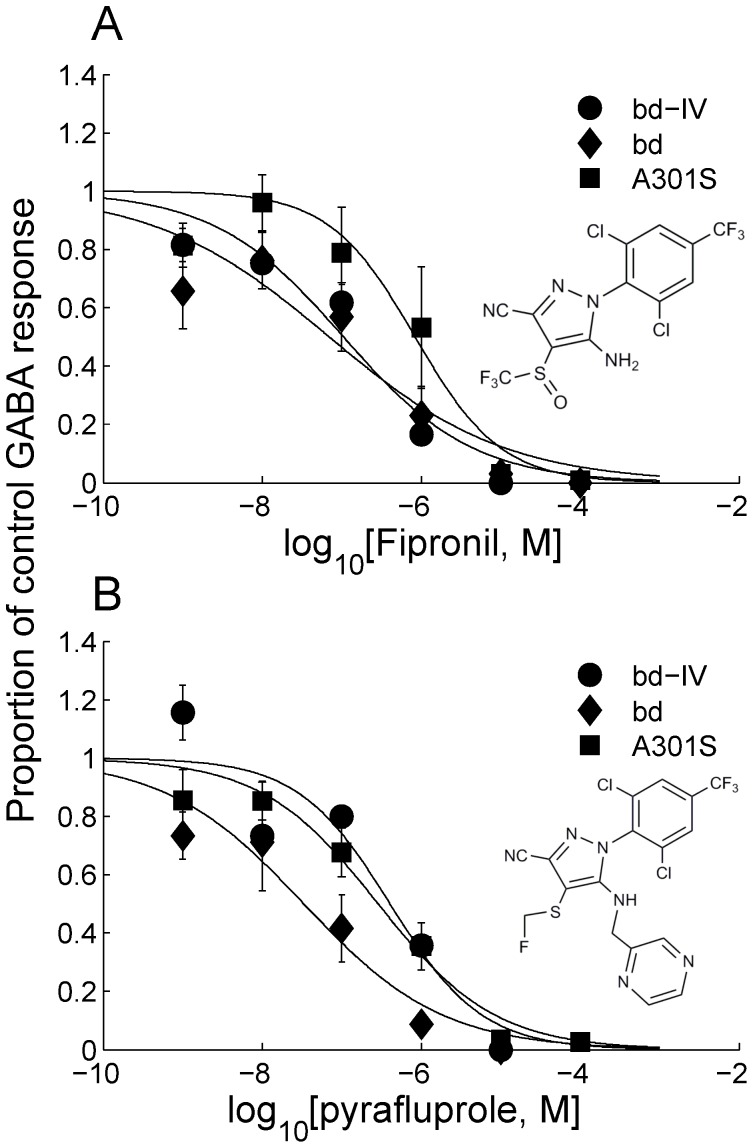
Concentration-inhibition curves showing responses elicited by the phenylpyrazoles (A) fipronil and (B) pyrafluprole to 1 mM GABA in wild-type, RDL_bd_IV and RDL_bd_IV/A^301^S. Each point represents the mean amplitude of the responses recorded in 2–13 oocytes from at least 3 separate batches. Structures of the two phenylpyrazoles are inserted.

When fipronil was applied alone (1 nM−100 µM) it induced no discernible current (data not shown). At concentrations of 10 nM to 100 µM, it reduced the response of the three receptor variants (RDL_bd_, RDL_bd_I/V, RDL_bd_I/VA^301^S) to GABA in a concentration-dependent manner (Fig. 4A). The pIC_50_s for fipronil on the 3 isoforms were RDL_bd_ = 6.48±0.13; RDL_bd_I/V = 6.20±0.44; RDL_bd_I/VA^301^S = 5.80±0.28. These values were not significantly different (F(2,55) = 1.20, P = 0.31). Whereas in the wild-type and I283V receptor, 1 µM fipronil effected in a 75% reduction of the response to 50 µM GABA, this was not achieved in the RDL_bd_I/VA^301^S receptor (50% reduction). Pyrafluprole also reduced the amplitude of GABA responses at concentrations of 10 nM to 100 µM (Fig. 4B). The estimated pIC_50_s were RDL_bd_ = 6.20±0.34; RDL_bd_I/V = 7.20±0.36; RDL_bd_I/VA^301^S = 6.21±0.16. These values were not significantly different one from another (F(2,27) = 2.46, P = 0.10). All 3 antagonists completely abolished GABA responses in all RDL isoforms at 100 µM.

**Figure 5:**
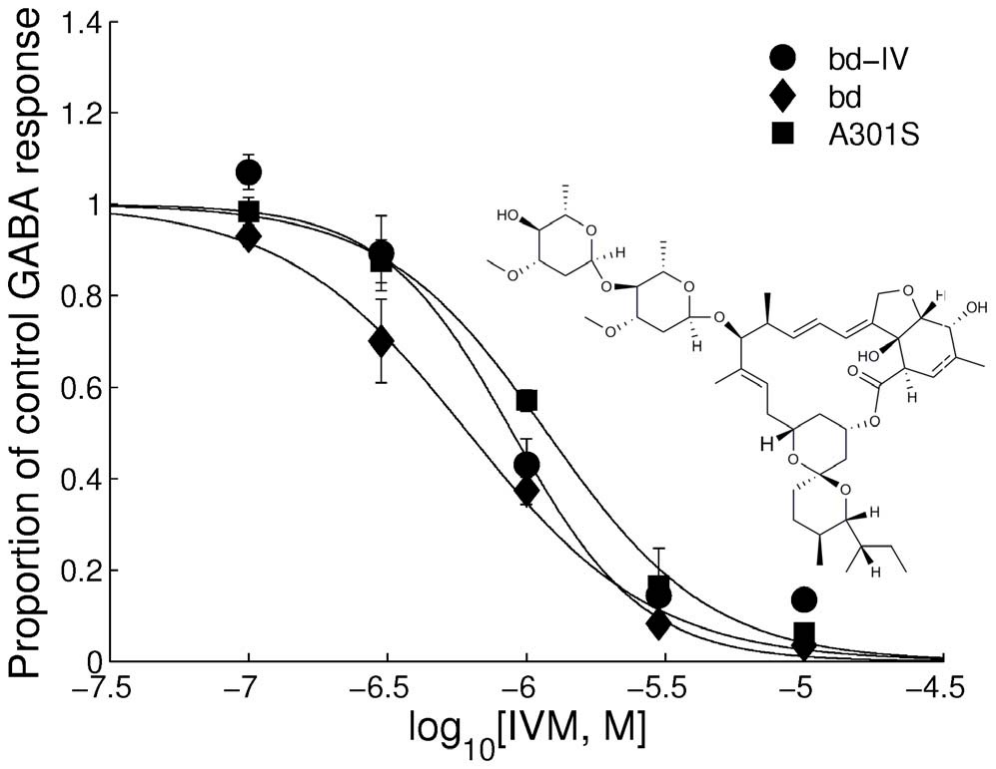
Effect of ivermectin on RDL_bd_. Concentration inhibition curves showing responses elicited by IVM to 100 µM GABA in wild-type, RDL_bd_IV and RDL_bd_IV/A^301^S. Each point represents the mean amplitude of the responses recorded in 2–6 oocytes from at least 3 separate batches. The structure of ivermectin is inserted.

All three RDL receptors showed no response to 10 µM IVM applied alone (data not shown). However, when co-applied with 100 µM GABA, the three RDL receptor variants showed a reduced response to GABA. When exposed to IVM, all three receptors produced a concentration-dependent suppression of amplitude to GABA (Fig. 5) with the following pIC_50_s = RDL_bd_ = 6.28±0.13, RDL_bd_I/V = 6.33±0.12 and RDL_bd_I/VA^301^S = 5.92±0.09. These values were significantly different one from another (F(2,51) = 5.13, P<0.001).

## Discussion

We have characterised aspects of the pharmacology of the most abundantly expressed isoform of *Drosophila* RDL variant RDL_bd_I/V, exploring for the first time the agonist profile and the actions of other important ligands including picrotoxin, fipronil and IVM. Jones et al. [18] found the RDL_bd_ splice variant to be the most abundant native variant in *D. melanogaster* and also found the single I283V edit to be the most prevalent isoform. I283V is located in the intracellular region close to the transmembrane region of TM1. That region of the subunit may contribute to the pathway of the permeating ion [36]. When compared with the RDL_bd_ wild-type, the addition of this edit had no significant effect on the EC_50_ of GABA. This was also observed by Jones et al. [18]. The RDL EC_50_ value recorded here is very similar to previously reported EC_50_ values of 56.9 µM [21] and 54 µM [18]. Here we found no statistically-significant effect of the addition of the A^301^S mutation on the GABA EC_50_. This is in contrast to Le Goff et al. [15], who detected a decrease in GABA EC_50_ from 70 µM to 26 µM with the addition of the A^301^G mutation in RDL_bd_R^122^G.

*Drosophila* RDL_ac_ and RDL_bd_I/V have a similar order of agonist potency [37], i.e. with GABA as the most potent agonist followed by muscimol, TACA, isoguvacine and lastly β-alanine. However, we did not see differences between the pEC_50_ values for GABA between the 3 different isoforms of RDL that we tested. In contrast, the EC_50_ values of agonists for the insect (Musca) bd type of receptors have been reported to be larger than those of RDL_bd_ [38] and *Dm* RDL_ac_ [39], although care is needed when comparing studies performed by different laboratories under different conditions and on different insect species. The alternative splicing of exon 6 (resulting in exons c or d) is likely to influence agonist potency as it generates diversity in loops C and F that contribute to the ligand-binding site [40].

From our docking calculations it appears likely that the five agonists studied bind to and activate RDL_bd_ through the same overall binding mode as suggested by Fig. 2H. A recent study of the human GABA_A_ receptor likewise suggests that GABA and muscimol bind with the same overall orientation [41], in a manner similar to our binding models. The lower potency of TACA and β-alanine relative to GABA might be explained by a lower binding affinity as predicted by the docking results (best affinities for GABA, TACA and β-alanine are −5.0 kcal/mol, −4.7 kcal/mol and −4.3 kcal/mol, respectively). The explanation for the lower potency of muscimol and isoguvacine relative to GABA on the other hand cannot be explained by the binding affinities predicted by the model (best affinity for muscimol is −5.2 kcal/mol and for isoguvacine either −5.5 kcal/mol for the less GABA like binding mode or −5.2 for the GABA like binding mode). The ring systems in both of these structures may interfere with some of the structural changes expected for the protein upon activation, however, the study of the dynamics is beyond the scope of this paper.

Insects showing high resistance to dieldrin also show reduced sensitivity to picrotoxin and the phenylpyrazoles, fipronil and pyrafluprole. This is also observed for RDL_bd_I/V, where the A^301^S mutation causes a 6-fold resistance to picrotoxin, 3-fold resistance to fipronil and 2-fold resistance to pyrafluprole compared to wild-type. Binding of picrotoxin in the pore region close to residues corresponding to A301 has been observed in the crystal structure of the *C. elegans* GluClα glutamate-gated chloride channel [24]. The addition of the I/V edit had no significant effect on the IC_50_s, which might be expected as I283V is located in TM1 and is not in contact with the pore-lining TM2 region where A^301^S is located.

IVM is known to interact with GABA receptors and several reports suggest that insect RDL and GluCl subunits co-assemble to form IVM-sensitive receptors [38], [42], [43]. A population of IVM-sensitive receptors is reported in *Drosophila* membranes containing *Dm*GluCl1α but lacking *Dm*RDL [42]. RDL_bd_ shows sensitivity to IVM when investigated as an antagonist. *Musca domestica* RDL_bd_ has also been investigated functionally [38], and the values obtained for its pEC_50_ (4.0) were similar to those obtained for Drosophila RDL_bd_ (4.24). However, the authors did not test for antagonist action of IVM.

It has been shown here that the I/V edit *per se* has no effect on IVM actions on RDL_bd_I/V, but the addition of the A^301^S mutation resulted in 2-fold resistance. This is similar to observations by Kane et al. [44] who observed that *Drosophila* carrying the RDLA^301^S mutation were 3.3-fold resistant to IVM compared to the wild-type. Studies on invertebrate ligand-gated chloride channels have identified several point mutations that affect their IVM sensitivity (See [45] for a recent review). Recent papers on different Cys-loop receptors from a variety of species have shown that amino acid residues in the TM2–TM3 loop and in transmembrane regions contribute to high affinity interactions between the receptor and allosteric ligands such as IVM [46]–[48]. Lynagh and Lynch [49] identified a single mutation, A288G, in the human glycine α1 receptor, increasing IVM sensitivity almost 100-fold while *Drosophila* carrying the P299S mutation in GluCl (located in the TM2–TM3 loop) are ten-fold less sensitive to IVM [44]. Modelling by Hibbs and Gouaux [24] showed that IVM makes contact with the TM2 pore-lining helix and the TM2–TM3 loop. In *C. elegans,* GluClα Ser260 located in TM2 hydrogen bonds with IVM. It is interesting to note that for other Cys-loop receptors, including the glycine and GABA_A_ receptors, that are directly activated by IVM [50], [51], there is also a serine at the equivalent position to S250 in GluClα.

We report the first pharmacological characterization of the predominantly expressed native splice and edit variant of the *Drosophila* GABA receptor (RDL_bd_I/V). The I/V edit does not impact greatly on the potency of GABA or the actions of GABA agonists. TACA and β-alanine are predicted from molecular modelling based on the *C. elegans* GluCl structure to bind with lower affinity than GABA explaining their lower potency in functional studies. The results for muscimol and isoguvacine are less readily explained structurally, and we are forced to speculate that it may relate to the efficiency with which these ligands induce the conformational change associated with the opening of the channel.

The A^301^S mutation reduced sensitivity to picrotoxin, fipronil and pyrafluprole but the I/V edit had no effect. The I/V edit did not impact on the blocking actions of IVM but the A^301^S mutation modified IVM IC_50_ values. We speculate that these effects are subtle modulations of the underlying dynamics and thus will require much more detailed exploration than is possible here.
